# Deworming programs for horses in the United States: past, present, and future

**DOI:** 10.1093/af/vfae018

**Published:** 2024-10-14

**Authors:** Jason L Turner

**Affiliations:** College of Agricultural, Consumer and Environmental Sciences, New Mexico State University, Las Cruces, NM 88003, USA

**Keywords:** anthelmintic resistance, deworming, equine, parasite

**Implications**

- The past: The development of a variety of effective, readily available, and easily administered equine paste dewormers in the 20th century revolutionized the practice of equine deworming and had a positive impact on equine health, but this has been accompanied by increased reports of AR in internal parasites.
- The present: Frequent reports of AR to the limited arsenal of anthelmintics available have raised awareness of the problem, and practices of horse owners appear to be changing by seeking more advice from veterinarians on deworming protocols.
- The future: New technologies from the field of genomics, gene editing, artificial intelligence, and data analytics may lead to the identification and development of new anthelmintic drugs or other control practices for internal parasites.

## Introduction

Developing deworming programs for horse farms based on scientific evidence has become more challenging as the concern over the development of anthelmintic resistance (AR) by internal parasites has continued to grow over the past few decades. The once simple recommendation of “deworm horses every two months” has become outdated and is strongly discouraged by many experts in the field as it is believed to hasten the development of AR in parasites which renders commonly used deworming drugs less effective. This perspective reviews historical practices of the past, the present situation, and the hope for future innovations in the field stemming from new technologies.

## The Past

The health risks posed by internal parasites of the horse have been a concern of owners and caretakers over the centuries. Prior to the development and use of modern anthelmintic drugs in equine management, the horse’s own immune system was the primary means of controlling these foreign invaders. Immunity to internal parasites involves both innate and acquired immune responses to deal with the infection. Horses less than 3 years old do not have the protective level of immunity to some parasites as that acquired by horses of age three and over.

Early veterinary treatments for internal parasites included such things as hens’ eggs, animal feces, a variety of herbs, plants, and their extracts as well as other chemicals which often had severe side effects for the horse. The mid-20th century saw the development of a variety of chemicals to be tested and used as anthelmintics in horses. In 1966 the practice of controlling large strongyles using a treatment interval of 6 to 8 weeks between deworming was recommended (reviewed by Lyons et al., 1999). Much like the “Green Revolution” that changed global crop production, these new chemical anthelmintics when provided in an easy-to-administer paste formulation in the 1970s, revolutionized the approach to equine deworming by taking the procedure out of the hands of the equine veterinarian that previously had been called to administer “chemical cocktails” by nasogastric tube to the horse. The readily available, and often used, tube dewormer brought about improvements in equine health. Yet, “too much of a good thing can be a bad thing” as was observed when interval dosing regimens likely contributed to the development of AR in these parasites to the chemicals previously used to control them. In general, the interval from a new dewormer first being released on the market to published scientific reports of AR being observed ranged from approximately 5 to 25 years depending on the specific drug.

The industry’s heavy reliance on nonselective chemical methods of control, rather than diagnostic-based deworming programs, for equine parasites is illustrated in survey data. The National Animal Health Monitoring Systems (NAHMS) Equine 2005 Study NAHMS (2007) reported the percentage of equine operations that performed fecal egg count (FEC) tests for parasites during the previous year decreased from 20% in 1998 to 13.5% in 2005. The NAHMS Equine 2015 Study NAHMS (2017) reported that in the majority of operations surveyed (72.9%), a veterinarian had never recommended the FEC as part of the farm’s deworming program.

## The Present

Since the release of moxidectin for use in horses in the USA in 1997, there have been no other new products added to the arsenal of broad-spectrum anthelmintics. With the goal of maintaining the effectiveness of the anthelmintics available, experts in the field have focused on more judicious use of deworming agents based on selective treatment of individuals with a higher parasite burden as determined by an FEC test.

**Figure 1.**
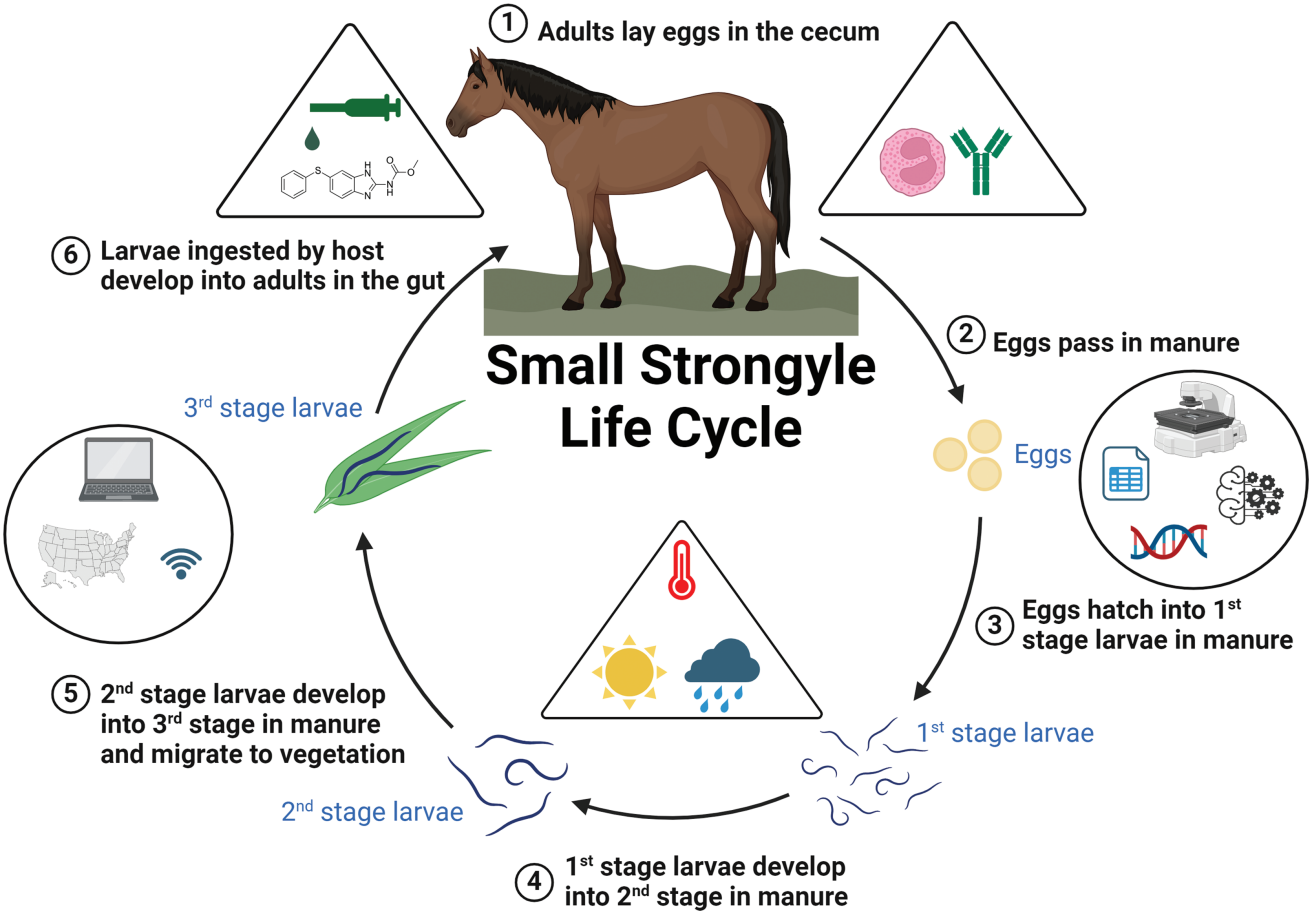
Overview of the life cycle of the small strongyle infecting horses. Icons in triangles represent known factors involved in parasite control. Icons in circles represent technologies that may lead to innovation in control methods. Created with BioRender.com.

While ascarids, bots, pinworms, tapeworms, and threadworms are commonly found in horses, small strongyles are the most prevalent internal parasite of horses. To complement the chemical control of internal parasites, recommendations have also sought to use environmental conditions in the free-living (outside of the horse) stages of internal parasites, such as small strongyles (Figure 1), to minimize the transmission of parasites by reducing the number of parasites found on pastures. Recent studies using computer modeling to study the impact of climate on AR (Nielsen et al., 2019) and the use of gene sequencing to identify parasite species in various ages of horses from different climate zones (Abbas et al., 2023) provide evidence of the important role that climate factors play in modern deworming strategies.

A challenge in the horse world is that of overcoming long-held traditions. The adoption of “deworm horses every 2 months” as a management practice was widely used for many years. Despite recommendations suggesting more limited use of dewormers for nearly two decades, this method is still widely employed. However, the 2021 American Horse Publications Equine Industry Survey (AHP, 2021) reported the following positive changes in horse owners: 54.4% reported their veterinarian is involved in developing their horses’ deworming program, 60% stated their veterinarian recommended an FEC test, and the number of owners deworming 1 to 3 times per year increased while the number of owners deworming up to 6 times per year decreased.

With this change in mindset and return to involving the veterinarian in deworming decisions, perhaps the rise in AR reports can be curbed and the effectiveness of the primary anthelmintic agents available can be preserved. Another challenge in this area has been differences in FEC test methodology, and determination of AR, making it difficult to compare results between studies. Recently the World Association for the Advancement of Veterinary Parasitology (Nielsen et al., 2022) published guidelines for evaluating the efficacy of equine anthelmintics which recommends standardized procedures for investigators in this field. This standardized approach should allow for greater predictive inference when evaluating the results of separate research trials in the future.

## The Future

The standardization of research protocols and the change in horse owner practices bode well for maintaining the effectiveness of the anthelmintic drugs currently available. Climate factors by month of the year, parasite species prevalence and susceptibility to specific anthelmintics, and incidence of AR for a specific drug with respect to geographic location provide supplementary data that can be used along with horse-specific information, such as age, previous FEC, and treatments, in predictive analytics that may offer better parasite control strategies based on that information. Advances in artificial intelligence and machine learning already at hand will increase the volume of FEC tests that can be conducted. Even now, some animal health companies have expanded their diagnostic services to include such offerings, and the integration of those individual FEC test results in models with the data mentioned above may yield individualized treatment recommendations before the patient leaves the veterinarian’s clinic. The use of data analytics could help control the spread of AR-positive parasites from one geographic area to other areas across the country by using risk maps or similar information-sharing methods.

Advances in artificial intelligence coupled with “omics” data provide another approach for screening new drugs, or repurposing existing drugs, for anthelmintic use in horses. Genomic studies may reveal specific genes that make some horses more resistant to parasites than others. Genomics may also identify specific target sequences in parasites that are AR, and this may aid in the development of new anthelmintic drugs or other treatments for those populations. Furthermore, crystal proteins from *Bacillus thuringiensis* (Chicca et al., 2022), condensed tannins and other compounds from plants (Grimm et al., 2022), and extracts from essential oils (Trailovic et al., 2021) show some promise as novel anthelmintic agents that could supplement the current arsenal in various ways.

In summary, the future holds great promise for the field of equine parasitology. While further study is warranted, so is credit to the multi-disciplinary and international collaboration among the scientists who are accomplishing this important work.

## About the Authors

 Jason L. Turner is the Horse Specialist for the Cooperative Extension Service in New Mexico. He has contributed to the teaching, research, and Extension mission of New Mexico State University since 2002. He provides research-based information and advice on a variety of issues to a diverse population of horse owners across the state, and he has served in leadership roles on the Board of Directors for many professional societies and industry associations at the local, state, and national levels. His graduate training studied assisted reproductive technology, immunology, and animal health. Current research interests include equine health management and novel approaches to equine owner education. **Corresponding author:**jlturner@nmsu.edu
